# Effect of Changes in Surgical Strategies for the Treatment of Primary Rhegmatogenous Retinal Detachment on Functional and Anatomical Outcomes: A Retrospective Analysis of 812 Cases from the Years 2004 to 2012

**DOI:** 10.3390/jcm12062278

**Published:** 2023-03-15

**Authors:** Aleksandra Sedova, Christoph Scholda, Thomas Huebl, Irene Steiner, Stefan Sacu, Michael Georgopoulos, Ursula Schmidt-Erfurth, Andreas Pollreisz

**Affiliations:** 1 Department of Ophthalmology and Optometry, Medical University of Vienna, 1090 Vienna, Austria; 2 Center for Medical Data Science (CeDAS), Institute of Medical Statistics, Medical University of Vienna, 1090 Vienna, Austria

**Keywords:** primary rhegmatogenous retinal detachment, retinal detachment surgery outcomes, scleral buckling, vitrectomy, combined vitrectomy/scleral buckling

## Abstract

Background: At the Department of Ophthalmology and Optometry at the MUV surgical method (scleral buckling, vitrectomy, combined vitrectomy/scleral buckling) and timing (daytime, nighttime) for the treatment of primary rhegmatogenous retinal detachment (RRD) changed continuously in the years 2004 to 2012. This study aims to evaluate changes in surgical strategies over time including their impact on functional and anatomical outcomes. Methods: Retrospective evaluation of patients operated on primary RRD between the years 2004 and 2012. Baseline demographic data, month 3 best-corrected visual acuity (BCVA), surgical method, single success surgery, surgical timing, and intraoperative complications were analyzed. Results: Overall, 812 eyes of 812 patients with a mean (±SD) age of 58.1 ± 13.3 years were included. A total of 413 (51%) patients presented with macula-on and 359 (44%) with macula-off RRD. Month 3 BCVA increased over time, both in macula-on or macula-off groups (*p* < 0.001). The rate of complete retinal reattachment 3 months postoperatively increased significantly from 65% in 2004 to 83% in 2012 in both groups. Scleral buckling surgeries decreased continuously from 95% to 16% with an appropriate increase in vitrectomies as well as a decrease in surgeries during nighttime (68% in 2004, 6% in 2012) with equal or better visual and functional outcomes. Conclusion: Our data showed that improving functional and single-success surgery outcomes in patients operated on for primary RRD. In the years 2004 to 2012, surgical techniques shifted from scleral buckling to primary vitrectomy and were increasingly scheduled during the daytime.

## 1. Introduction

Rhegmatogenous retinal detachment (RRD) is the most commonly occurring form of retinal detachment with the incidence ranging between 6.3 and 17.9 cases per 100,000 subjects per year [1]. It is defined as a pathological accumulation of fluid between the neurosensory retina and the retinal pigment epithelium caused by retinal tears and tractional forces allowing fluid influx [2]. RRD is a vision-threatening condition mainly affecting individuals over 50 years old and requiring retinal reattachment surgery [3]. Men are 1.3 to 2.3 times more likely to be diagnosed with RRD [1]. Timing of a surgical intervention is of uppermost importance for the visual outcome and is also dependent on the presence of macular detachment [4,5]. In cases with macula-off RRD, detachment of the macula causes photoreceptor cell death through apoptosis, which is responsible for poor visual outcomes [6]. Cells undergoing apoptosis are already found within 24 h following RRD, reaching a peak at 2 days and then dropping gradually over the following 5 days [6].

There are different methods for surgical repair available, including pars plana vitrectomy, scleral buckling, and pneumatic retinopexy [7,8]. Scleral buckling was first introduced by Custodis in 1949, widely popularized by Charles Schepens in the 1950s, and improved by Lincoff in the 1960s [9,10]. In the 1970s, pars plana vitrectomy was developed by Robert Machemer and proved to be equally effective as scleral buckling for the management of RRD [11,12,13]. In 1986, pneumatic retinopexy was firstly proposed by Hilton and Grizzard as a procedure for selected RRD cases, for which hospitalization is not needed [14]. All these techniques have undergone significant improvements since their introduction, with vitrectomy being the most commonly used surgical approach to date [15].

The aim of this retrospective study was to evaluate the effects of surgical method (scleral buckling vs. vitrectomy) and scheduling of surgery (routine daytime program vs. emergency surgery outside routine program including nighttime) on functional and anatomical outcomes in eyes with primary rhegmatogenous retinal detachment in a single tertiary referral center.

## 2. Materials and Methods

### 2.1. Subjects

This retrospective study was conducted at the Department of Ophthalmology and Optometry at the Medical University of Vienna (MUV) according to the Declaration of Helsinki, including current revisions and Good Clinical Practice (GCP) guidelines. The study protocol was approved by the Ethics Committee of MUV (Ethics number: EK1766/2016). Clinical records of all eyes treated for primary rhegmatogenous retinal detachment with either encircling scleral buckling, segmental scleral buckling or vitrectomy between the years 2004 and 2012 were reviewed by a vitreoretinal surgeon (A.P.). Exclusion criteria included cases with symptoms of retinal detachment for more than 4 weeks (due to expected inferior visual, functional, and morphological outcomes in macula-off patients) history of any prior vitreoretinal surgery, proliferative vitreoretinopathy stage C or higher, giant retinal tear, traumatic or post-traumatic retinal detachment, endophthalmitis or uveitis in the case history, retinoschisis-related detachment, and presence of retinal diseases (diabetic retinopathy, age-related macular degeneration, epiretinal membrane, macular hole), contralateral eye already presented with retinal detachment, incomplete data in the patient charts, follow-up visits less than 2 months after surgery (some patients prefer having their follow-up visits at their local eye center for travel distance reasons). Data collected included age, sex, surgical method, duration of surgery, presence of macula detachment (macula-off), preoperative and postoperative (3 months postoperatively) Snellen best corrected visual acuity (BCVA), intraoperative complications, lens status, single surgery anatomical outcome success, and timepoint of surgical intervention (daytime, nighttime). Single surgery anatomical success was defined as a complete attachment of the retina 2–3 months postoperatively after a single procedure. For analysis, the patients were divided into two groups depending on the presence of macula detachment (macula-on and macula-off groups). Further, surgeons were divided into two groups depending on their specialization: vitreoretinal surgery specialist and general ophthalmic surgeon. A total of 198 patients did not show up for their scheduled follow-up visit 3 months post-surgery. Re-surgeries were excluded from the analyses.

### 2.2. Surgical Procedure

The surgeries were performed by a total of 13 vitreoretinal surgery specialists and 17 general ophthalmic surgeons with all procedures done under general anesthesia. After disinfection of eyelids and periorbital skin with 5% polyvidone-iodine solution, polyvidone-iodine eye drops were applied at the beginning and at the end of surgery.

A 23-gauge (*n* = 306, 78%) or 20-gauge (*n* = 88, 22%) pars plana vitrectomy was performed with the OS3 surgery system (Oertli Instruments, Berneck, Switzerland) using peristaltic pump settings and a single-use pneumatic stripper (SPS) by all surgeons. For the 23-gauge system valved Oertli trocars were used, while for the 20-gauge system a trocar-free approach was applied. Every patient received a complete vitrectomy with cryo- or endolaser coagulation and gas (SF6, C2F6, or C3F8) or silicone oil filling.

The standard procedure for the scleral buckling procedure is as follows. After the opening of the conjunctiva at the limbus, the retinal tear was coagulated with cryocoagulation under indirect ophthalmoscopy. A scleral explant (radial or circumferential sponge, encircling silicone band) was fixated with 4-0 mersilene sutures. Subretinal drainage was performed based on the surgeon’s discretion. The conjunctiva was closed with 7-0 vicryl sutures. In some cases, the vitrectomy procedure was combined with scleral buckling.

For analysis, the time of surgery was divided into a routine daytime program, which included surgeries performed between 7 am and 4 pm and an outside routine program encompassing surgeries between 4 pm and 7 am.

### 2.3. Statistical Analysis

Quantitative variables are reported as mean ± standard deviation. For qualitative variables, absolute frequencies and percentages are reported.

To analyze if BCVA at baseline changed over time, a univariate linear model was calculated with the year (2004–2012) as the independent variable for the entire sample and the two subgroups (macula-on and macula-off).

The effect of surgeon specialization (general ophthalmic surgeon/VR surgery specialist), scheduling of surgery (routine surgical program/outside routine surgical program), surgical method (vitrectomy/buckle/vitrectomy + buckle surgery), and year of surgery (2004–2012) on BCVA at month 3, was analyzed by linear models adjusted for BCVA at baseline. Additionally, a linear model with stepwise variable selection using the AIC criterion was calculated (R-function step), including all the independent variables mentioned above, whereby the interaction between year (2004–2012) and surgery before 2009 (yes/no) was additionally included as an independent variable. The null hypothesis of the interaction term is that the slope of the regression line does not differ between the years 2004–2008 and 2009–2012. The final model with the selected variables was then calculated using all valid observations. If the F-test of the surgical method revealed a *p*-value < 0.05, pairwise comparisons were conducted. The dichotomous endpoints “single success surgery” and “intraoperative complications” were analyzed analogously by calculating logistic regression models. Due to the small number of patients with intraoperative complications, no multivariable logistic regression model was calculated for this endpoint.

Statistical analyses were carried out with R 4.0.5. For all analyses, the significance level has been set to 0.05. Estimates are reported with 95% confidence limits. No adjustment for multiple testing was done. Hence, the interpretation of the *p*-values is descriptive.

## 3. Results

Eight-hundred and twelve eyes from 812 patients receiving surgical treatment for rhegmatogenous retinal detachment between the years 2004 and 2012 were eligible for inclusion in this retrospective study. The mean participant age at diagnosis was 58.1 ± 13.3. Out of these patients, 294 were female (36%). A total of 502 eyes (62%) were phakic, 294 eyes (36%) pseudophakic, and 16 eyes (2%) aphakic. Out of all operated eyes, 433 (53%) were right eyes. In addition, 413 (51%) patients presented with macula-on retinal detachment, and 359 (44%) patients with macula-off. In the surgical records of 40 (5%) patients, there was no indication regarding macula status. The number of macula-on patients was 45, 48, 45, 50, 40, 45, 51, 40, and 49, respectively in the 9 years ranging from 2004 to 2012. The number of macula-off patients was 27, 30, 37, 36, 48, 57, 43, 32, and 49, respectively analyzed in the same time period.

### 3.1. Choice of Surgical Method over Time

**Figure 1:**
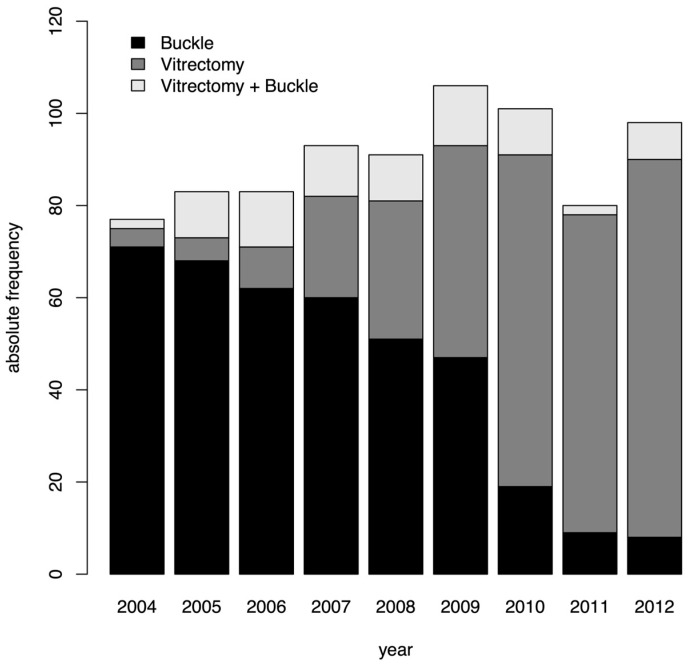
A bar chart showing the increasing number of performed vitrectomies (dark grey) in the years 2004 to 2012 with a simultaneous decrease of scleral buckle surgeries (black). The number of combined procedures (vitrectomy + scleral buckle) is shown in light grey.

The number of performed vitrectomies increased continuously from 2004 to 2012 with an appropriate decrease in scleral buckling surgeries (Figure 1). While in the year 2004, 5% of surgeries were performed with vitrectomy, this number increased to 84% 8 years later. The number of performed 20-gauge vitrectomies was 5, 9, 11, 8, 14, 10, 21, 4, and 6, respectively between the years 2004 and 2012. The number of 23-gauge vitrectomies was 0, 0, 7, 21, 22, 45, 60, 67, and 84, respectively during the same time period. Scleral buckling procedures decreased from 92% of all surgeries in 2004 to 8% in the year 2012 (number of performed buckles between 2004 and 2012: 71, 68, 62, 60, 51, 47, 19, 9, and 8, respectively). Combined procedures of vitrectomy and scleral buckling were performed in 2, 10, 12, 11, 10, 13, 10, 2, and 8 cases, respectively, between 2004 and 2012 with no trend regarding frequency observable over these years. See Table 1 for detailed information about surgery type and intraocular tamponade.

The number of performed vitrectomies was 5, 9, 18, 29, 36, 55, 81, 71, and 91 in the 9 years ranging from 2004 to 2012. The number of scleral buckle surgeries was 71, 74, 65, 63, 55, 51, 20, 9, and 7 in the same time period. Combined vitrectomy and scleral buckle procedures were performed in 2, 10, 12, 11, 10, 13, 10, 2, and 8 eyes during that time frame.

A total of 170 eyes (21%) required additional surgery due to not having an attached retina after the first surgery.

### 3.2. Scheduling of Surgery

In the years from 2008 to 2012, the majority of RD surgeries were performed during the routine surgical program (*n* = 81%), while between 2004 and 2007 only 43% of cases were scheduled during the daytime. In the year 2004, 68% of eyes were operated outside the routine program including nighttime, four years later this number dropped to 25%, decreasing further within the next 4 years and reaching 6% in 2012. In the years 2004 to 2008 about 58% of procedures during the routine surgical program were performed by surgeons specialized in vitreoretinal surgery. From 2009 onwards this number increased to over 96%.

See Table 2 for a detailed overview of performed surgeries according to group and surgeon specialisation.

The average time from the first presentation at the clinic to surgery increased from 0.5 to 1.48 days from 2004 to 2012.

### 3.3. Visual Acuity Outcomes

#### 3.3.1. Visual Acuity at Baseline

Among all macula-on and macula-off patients, BCVA at baseline did not change significantly over time (mean change per year [95% CI]: 0.00097 [−0.009; 0.011], *p* = 0.9). Within macula-off patients, the change of BCVA at baseline over time was statistically not significant either (mean change per year [95% CI]: 0.0044 [−0.00054; 0.0093], *p* = 0.08). Within macula-on patients, there was a borderline significant increase over time (0.011 [0.00005; 0.022], *p* = 0.050). Patients with unknown macula status at the time of surgery due to incomplete medical records were excluded from this analysis.

#### 3.3.2. Visual Acuity 3 Months Postoperatively in Macula-On Patients

**Figure 2:**
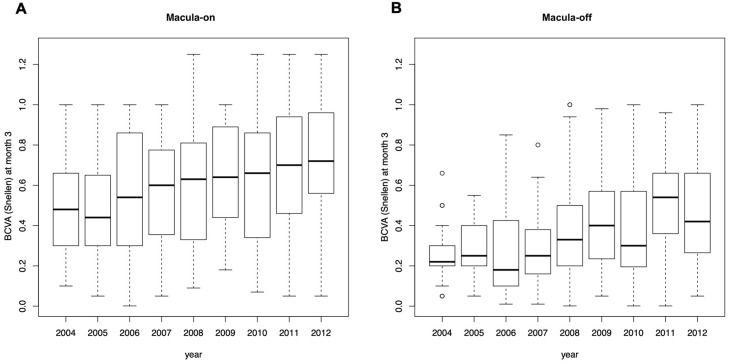
Box plots of BCVA (Snellen) at baseline and at month 3 in macula-on (**A**) and macula-off (**B**) patients grouped by year of surgery. In the box plots, the inferior boundary of the box indicates the 25th percentile, a black line within the box marks the median, and the superior boundary of the box indicates the 75th percentile. Outliers are defined as values that are smaller/greater than 1.5 times the interquartile range (IQR) from the box. Whiskers above and below the box indicate the minimum and the maximum, respectively, if no outliers are present. In the case of outliers, the whiskers extend to the smallest/largest value within the interval [25th percentile − 1.5 IQR; 75th percentile + 1.5 IQR].

In the macula-on patients, BCVA 3 months postoperatively significantly increased over time (mean change per year adjusted for visual acuity at baseline [95% CI]: 0.021 [0.009; 0.032], *p* = 0.0005; Figure 2A). BCVA 3 months postoperatively was on average significantly higher in patients after vitrectomy compared to scleral buckling (mean difference adjusted for visual acuity at baseline [95% CI]: 0.064 [0.002; 0.126], *p* = 0.04) and significantly lower in patients after combined vitrectomy/scleral buckling compared to vitrectomy (−0.142 [−0.264; −0.019], *p* = 0.02), whereas the difference between combined vitrectomy/scleral buckling vs. scleral buckling was not statistically significant (−0.078 [−0.197; 0.042], *p* = 0.2). Surgeons’ specialization in retinal surgery (0.02 [−0.04; 0.08], *p* = 0.5), scheduling of surgery (−0.02 [−0.08; 0.04], *p* = 0.5) had no significant effect on BCVA at month 3. In the stepwise linear regression model, only BCVA at baseline and year of surgery remained in the model.

#### 3.3.3. Visual Acuity 3 Months Postoperatively in Macula-Off Patients

In the macula-off patients, univariate analyses adjusted for BCVA at baseline revealed a significant effect on BCVA 3 months postoperatively of surgery year (0.027 [0.017; 0.038]. *p* < 0.0001; Figure 2B), surgeons’ specialization in retinal surgery (0.13 [0.07; 0.19], *p* < 0.0001) and surgical method (F-test: *p* < 0.0001), but not of the scheduling of surgery (−0.009 [−0.075; 0.058], *p* = 0.8). In the stepwise linear regression model, the variables BCVA at baseline (0.55 [0.33; 0.77], *p* < 0.0001), surgeons’ specialization in retinal surgery, surgery time, and surgical method were selected. BCVA 3 months postoperatively was higher for surgeries performed by surgeons specialized in retinal surgery compared to general ophthalmic surgeons (0.098 [0.032; 0.16], *p* = 0.004). Scheduling of surgery did not have any statistically significant effect on BCVA 3 months postoperatively (0.062 [−0.005; 0.13], *p* = 0.07). BCVA 3 months postoperatively was significantly higher in patients after vitrectomy compared to scleral buckling (0.115 [0.054; 0.177], *p* = 0.0003) and significantly lower after combined vitrectomy/scleral buckling compared to vitrectomy (−0.135 [−0.213; −0.058], *p* = 0.0007), whereas the comparison of combined vitrectomy/scleral buckling with scleral buckling was not statistically significant (−0.02 [−0.099; 0.059], *p* = 0.6).

### 3.4. Single Surgery Anatomical Success

The rate of complete retinal reattachment after a single procedure increased from 64% (46/72 eyes) in 2004 to 83% (81/98 eyes) in 2012 regardless of the surgical method. See Table 3 for detailed information. Patients with missing macula status in medical records at the time of surgery were excluded from this analysis.

#### 3.4.1. Single Surgery Anatomical Success in Macula-On Patients

In the univariate logistic regression models, surgeons’ specialization in retinal surgery (OR [95% CI]: 2.2 [1.42; 3.33], *p* = 0.0003), surgical method (F-test: *p* = 0.005), and year of surgery (OR [95% CI]: 1.15 [1.06; 1.25], *p* = 0.001) had a significant effect on single surgery success, but not scheduling of surgery (0.81 [0.53; 1.24], *p* = 0.3). The odds for single success surgery were higher in vitrectomy compared to scleral buckling (OR [95% CI]: 2.12 [1.34; 3.34], *p* = 0.001), whereas the comparisons with combined vitrectomy/scleral buckling were not statistically significant (vitrectomy + buckle vs. buckle: *p* = 0.25, vitrectomy + buckle vs. vitrectomy: *p* = 0.64). In the stepwise logistic regression model, surgeons’ specialization in retinal surgery and year of surgery remained in the model. According to this final model, the odds for single success surgery were 1.13 times higher (95% CI: [1.01; 1.26], *p* = 0.03) for surgeons specializing in retinal surgery compared to general ophthalmic surgeons and increased with increasing surgery year, but the effect of surgery years did not reach statistical significance (OR [95% CI]: 1.017 [0.997; 1.038], *p* = 0.1)

#### 3.4.2. Single Surgery Anatomical Success in Macula-Off Patients

Surgeons’ specialization in retinal surgery (2.4 [1.40; 3.98], *p* = 0.001), surgical method (F-test: *p* = 0.01), and year of surgery (1.17 [1.06; 1.29], *p* = 0.003), but not the scheduling of surgery (0.81 [0.45; 1.43], *p* = 0.5) had a significant effect on single surgery success. The odds for single surgery success were higher in patients after vitrectomy compared to scleral buckling (OR [95% CI]: 2.11 [1.24; 3.57], *p* = 0.006), but the comparisons with combined vitrectomy/scleral buckling were not statistically significant (vitrectomy + scleral buckling compared to scleral buckling: 2.02 [0.91; 4.5], *p* = 0.09, vitrectomy + scleral buckling compared to vitrectomy: 0.96 [0.42; 2.2], *p* = 0.9). In the stepwise logistic regression model, surgeons’ specialization in retinal surgery (OR [95% CI]: 1.13 [0.999; 1.27], *p* = 0.053) and surgery year (OR [95% CI]: 1.02 [0.996; 1.04], *p* = 0.1) remained in the model, but these variables failed to reach statistical significance.

### 3.5. Intraoperative Complications

Among macula-on and macula-off patients operated on in the years 2004–2008, intraoperative complications, such as vitreous hemorrhage, iatrogenic retinal defects, choroidal hemorrhage, displacement of infusion cannula, and scleral perforation when placing scleral sutures occurred in 24 out of 406 eyes (5.9%). Among macula-on and macula-off patients operated on in the years 2009–2012, intraoperative complications occurred in 16 out of 366 eyes (4.4%). In the macula-on patients as well as in macula-off patients, intraoperative complications did not change significantly over time (*p* = 0.5/*p* = 0.4). In the macula-on/macula-off groups, surgeons’ specialization in retinal surgery (*p* = 0.7/*p* = 0.2), surgical method (*p* = 0.5/*p* = 0.6), scheduling of surgery (*p* = 0.3/*p* = 0.9) had no significant effect on intraoperative complications. Patients with missing information in the medical records regarding macula status at the time of surgery were not included in this analysis.

## 4. Discussion

In our study, we evaluated changes in surgical strategies for the treatment of RRD and their impact on functional and anatomical outcomes. Between 2004 and 2012 we observed a shift in surgical methods from scleral buckling towards vitrectomy as well as in surgical scheduling towards a routine daytime program. These changes in treatment strategies led to an improvement in functional and single-surgery success outcome.

In 2008, a new strategy for the treatment of RRD was implemented in our department. The modified approach called for surgery with a delay of no longer than 24 h for macula-on and 48 h for patients presenting with macula-off RRD. This strategy provided better planning and an opportunity to perform surgery during a routine daytime program preferably by vitreoretinal surgery specialists.

The macula status at the time of surgery is the main prognostic factor for visual outcome. Several studies have shown visual prognosis to be directly correlated with the duration of macula-off RRD, while others demonstrated that a surgery delay of up to 7–8 days does not affect visual results [4,16,17,18,19,20]. In cases with an attached macula, a timely surgical intervention is recommended in order to prevent detachment of the macula with worse visual outcomes [5,21]. Ehrlich et al. showed in a retrospective study of patients with macula-on RRD treated with small-gauge vitrectomy, that no statistically significant correlation was found between time to surgery and anatomical and visual outcomes. However, 83% of patients were operated on within 24 h of their presentation at the clinic. Similar results among patients with macula-on RRD yet managed with scleral buckling (56% of subjects treated within 24 h) were reported by Wykoff et al. [22]. In a prospective study by Ho et al. analyzing eyes with macula-on RRD, a shift of subretinal fluid towards the macula in 13% of eyes at the time of surgery was observed, leading to macular detachment only in 4% of eyes (2 cases within 24 h, 1 case at day 4) [23]. In this study, slightly over 60% of cases were treated within 24 h and 26% of surgeries were performed outside normal working hours [23]. These studies support our results that a minimal delay of the surgery (maximum 24 h for macula-on cases) does not worsen functional and visual outcomes.

Surgery for RRD performed as an emergency after regularly planned cases or during nighttime has an impact on the performance of staff due to fatigue, which may lead to higher complication rates [24]. Furthermore, having a surgical team available and keeping the facilities running during night hours usually results in higher costs [25].

In general, macula-on RRD has a better visual prognosis compared to macula-off RDD [26]. In our study, mean BCVA 3 months postoperatively improved significantly from 2004 to 2012, both in subjects with macula-on or macula-off detachments. Among macula-off patients treated after 2008, the mean BCVA 3 months postoperatively was significantly higher in the vitrectomy group compared to the scleral buckling group. Also, macula-on patients operated on after the year 2008 showed better visual outcomes in the vitrectomy group, but these changes were not statistically significant. In a prospective study with 199 macula-on patients treated with scleral buckling, Wykoff et al. showed that the median BCVA two months postoperatively was similar (0.67) to the one observed among our patients. In a retrospective study by Bourla et al., analyzing 25 patients with vitrectomy operations, the mean final BCVA was 0.7 in the macula-on group and 0.3 in the macula-off group, which corresponds to our results [27]. In our study, there was a minimal decrease in the mean BCVA in macula-on patients from baseline, which is clinically not significant and potentially caused by cataract formation. In the medical charts reviewed, cataract grades were not recorded and cataract surgeries were often performed in other centers with no access to these data.

In a large prospective study on 291 subjects with macula-off RRD, two thirds of patients demonstrated final BCVA to be over 0.33 Snellen [16]. Among these subjects, 77% were treated with vitrectomy. Another prospective study conducted by Heimann et al. showed a statistically significantly better final BCVA in the scleral buckling group (0.5 Snellen) compared to the vitrectomy group (0.33 Snellen) among phakic patients. Upon taking into account pseudophakic/aphakic patients, there was no difference in BCVA improvement between both treatment groups [28]. A systematic Cochrane Review comparing vitrectomy and scleral buckling in the treatment of RRD published in 2019 revealed similar outcomes regarding visual acuity [29].

In our study, the rate of complete retinal attachment 2–3 months postoperatively after a single procedure increased significantly over time from 65% in 2004 to 83% in 2012 in all subjects regardless of the treatment method. Single surgery success rates also improved regardless of macula-on or off status after 2008. Heimann et al. could show significantly better primary anatomical success rates in pseudophakic/aphakic patients treated with vitrectomy compared to the ones managed with buckling (72 vs. 53%) [28]. Another prospective study showed 69% single surgery anatomical success rates at month 6 in pseudophakic eyes treated with vitrectomy [30], while Bourla et al. demonstrated 98% single surgery success rates with vitrectomy [27]. Wykoff et al. reported primary anatomic success rates of 88% among macula-on patients operated with scleral buckling [22]. This rate is higher compared to our results (60% before 2008 and 78% after 2008). A potential explanation for the lower rates in our scleral buckling cohort could be that the majority of the procedures were performed during nighttime by general ophthalmic surgeons not specialized in retinal detachment cases. A recent systematic Cochrane Review conducted by Znaor et al. concluded that there is no difference between vitrectomy and scleral buckling in terms of final anatomical success rates [29]. Overall, the shift of surgeries for RRD in our clinic towards optimized surgical settings during the daytime is associated with reduced re-detachment rates.

There are several limitations to this study. First, the study was retrospective in design and therefore factors such as amblyopia, location and number of retinal holes, and the differentiation between PVR grades A and B were not reliably evaluable in the medical records. Secondly, from 2009 onwards there were fewer general ophthalmic surgeons performing retinal detachment surgeries. Thirdly, due to the grouping together of surgeons in our analyses, study outcomes may have been affected by different surgical skills and experience. Fourthly, due to the short follow-up period, there is a possibility of a missed retinal reattachment more than 3 months postoperatively if the patient did not visit our clinic. The strengths of this retrospective study are the number of patients included and the single center setting with uniform surgical systems used by the surgeons.

## 5. Conclusions

In conclusion, our retrospective analysis showed continuous improving anatomical and functional outcomes in patients treated for primary macula-on or off RRD when shifting surgical methods from scleral buckling to vitrectomy and scheduling of surgeries preferentially in the routine daytime program.

## Figures and Tables

**Table 1:** The number of performed procedures between the years 2004 and 2012 grouped according to the surgical method and gas/silicone oil filling (*n* = number of surgeries per year; % = percentage per year).

Year	Vitrectomy		Scleral Buckling	Combined Vitrectomy/Scleral Buckling	
	Gas	Silicone Oil		Gas	Silicone Oil
2004	*n* = 0 (0%)	*n* = 4 (5%)	*n* = 71 (92%)	*n* = 1 (1%)	*n* = 1 (1%)
2005	*n* = 5 (6%)	*n* = 0 (0%)	*n* = 68 (82%)	*n* = 3 (4%)	*n* = 7 (8%)
2006	*n* = 9 (11%)	*n* = 0 (0%)	*n* = 62 (75%)	*n* = 8 (10%)	*n* = 4 (5%)
2007	*n* = 20 (22%)	*n* = 2 (2%)	*n* = 60 (65%)	*n* = 7 (8%)	*n* = 4 (4%)
2008	*n* = 25 (27%)	*n* = 5 (5%)	*n* = 51 (56%)	*n* = 8 (9%)	*n* = 2 (2%)
2009	*n* = 45 (42%)	*n* = 1 (1%)	*n* = 47 (44%)	*n* = 12 (11%)	*n* = 1 (1%)
2010	*n* = 70 (69%)	*n* = 2 (2%)	*n* = 19 (19%)	*n* = 8 (8%)	*n* = 2 (2%)
2011	*n* = 68 (85%)	*n* = 1 (1%)	*n* = 9 (11%)	*n* = 2 (2%)	*n* = 0 (0%)
2012	*n* = 79 (81%)	*n* = 3 (3%)	*n* = 8 (8%)	*n* = 6 (6%)	*n* = 2 (2%)

**Table 2:** The number of surgeries performed between the years 2004 and 2012 grouped according to time of surgery and surgeon specialization. *n* = number of surgeries per year; % = percentage per year.

Year	Routine Program (7 a.m.–4 p.m.)						Outside Routine Program (4 p.m.–7 a.m.)					
	All Surgeons		Vitreoretinal Surgery Specialists		General Ophthalmic Surgeons		All Surgeons		Vitreoretinal Surgery Specialists		General Ophthalmic Surgeons	
	*n*	% of All Surgeries	*n*	% of Surgeries in Routine Program	*n*	% of Surgeries in Routine Program	*n*	% of All Surgeries	*n*	% of Surgeries Outside Routine Program	*n*	% of Surgeries Outside Routine Program
2004	24	32	10	42	14	58	52	68	16	31	36	69
2005	34	41	10	29	24	71	49	59	14	29	35	71
2006	39	47	19	49	20	51	44	53	16	36	28	64
2007	49	53	34	69	15	31	44	47	18	41	26	59
2008	68	75	51	75	17	25	23	25	12	52	11	48
2009	80	79	75	94	5	6	21	21	10	48	11	52
2010	83	82	80	96	3	4	18	18	14	78	4	22
2011	67	86	67	100	0	0	11	14	11	100	0	0
2012	89	94	86	97	3	3	6	6	6	100	0	0

**Table 3:** The number of single surgery anatomical success surgeries from 2004 to 2012 grouped according to macula status and surgical method (*n* = number of surgeries with single surgery anatomical success per year; % = percentage per year). Patients with missing macula status are not included.

Year	Macula-On	Macula-Off	Vitrectomy	Scleral Buckling	Combined Vitrectomy/Scleral Buckling
2004	*n* = 27 (60%)	*n* = 19 (70%)	*n* = 3 (100%)	*n* = 41 (61%)	*n* = 2 (100%)
2005	*n* = 26 (54%)	*n* = 20 (67%)	*n* = 1 (20%)	*n* = 38 (59%)	*n* = 7 (78%)
2006	*n* = 29 (64%)	*n* = 25 (68%)	*n* = 8 (89%)	*n* = 38 (62%)	*n* = 8 (67%)
2007	*n* = 32 (64%)	*n* = 23 (64%)	*n* = 13 (72%)	*n* = 37 (64%)	*n* = 5 (50%)
2008	*n* = 28 (70%)	*n* = 36 (75%)	*n* = 19 (66%)	*n* = 36 (72%)	*n* = 9 (100%)
2009	*n* = 31 (69%)	*n* = 45 (79%)	*n* = 34 (77%)	*n* = 31 (67%)	*n* = 11 (92%)
2010	*n* = 39 (76%)	*n* = 37 (86%)	*n* = 54 (82%)	*n* = 13 (72%)	*n* = 9 (90%)
2011	*n* = 32 (80%)	*n* = 25 (78%)	*n* = 51 (82%)	*n* = 5 (62%)	*n* = 1 (50%)
2012	*n* = 38 (78%)	*n* = 43 (88%)	*n* = 70 (85%)	*n* = 5 (62%)	*n* = 6 (75%)

## Data Availability

The datasets generated during and/or analyzed during the current study are available from the corresponding author on reasonable request.
